# Gonnorrhoeal Infection of the Mucous Membrane of the Mouth in New-born Infants

**Published:** 1893-05

**Authors:** 


### Selections and Abstracts.

---

GONORRHŒAL INFECTION OF THE MUCOUS MEMBRANE OF THE MOUTH IN NEW-BORN INFANTS.—From the study of five cases of gonorrhœal infection of the mouth in the *Konigsberg Obsterical Clinic*, Dr. Rosinsky has drawn the following picture of the disease: Without preceding inflammatory redness, a white discoloration appears upon the anterior two-thirds of the tongue, the tongue, the plaques of Bednar, the hamulus pterygoideus, and along the ligamentum pterygomandibularum in the lower jaw, finally upon the front parts of the gums. After twenty-four to thirty-six hours the color becomes yellow. The patches elevate themselves plateau-like over the surrounding tissues, and their surfaces are raw. The superficial epithelium forms with extravasated pus-cells, a thick layer, resembling the scrapings from the cut surface of a septic spleen. On the third day the regeneration of the epithelium begins; this is marked by an inflammatory redness around the edge of the patch. Healing follows without treatment, in an ideal manner, no trace of scar or discoloration remaining. From the microscopical examination of some excised tissue, Rosinky has gleaned the following: The gonococci were never found, in stained sections, intra-cellular. They were seldom found intra-cllular in the superficial flakes. Gonococci cannot penetrate into the body of healthy, living cells; they accomplish this only when the single cells are cut off from the conditions of life. In the connective tissue gonococci invasion was found. Rosinsky believes this to be typical pure gonorrhœal inflammation of the mucous membrane. The relatively infrequent gonorrhœal inflammation of the mucous membrane of the mouth in adults, in contradistinction to infants, he believes to be due to the tenderness of the epithelium of the mouth in the new-born.—*Annals of Gynæcology.*

